# Optogenetic tools controlled by ultraviolet-B light

**DOI:** 10.1007/s42994-021-00049-y

**Published:** 2021-05-21

**Authors:** Xinhao Ouyang, Hui Ren, Xi Huang

**Affiliations:** State Key Laboratory of Cellular Stress Biology, School of Life Sciences, Xiamen University, Xiamen, 361102 China

**Keywords:** Optogenetics, UV-B light, UVR8, COP1

## Abstract

Decades of genetic, molecular and biochemical studies in plants have provided foundational knowledge about light sensory proteins and led to their application in synthetic biology. Optogenetic tools take advantage of the light switchable activity of plant photoreceptors to control intracellular signaling pathways. The recent discovery of the UV-B photoreceptor UV RESISTANCE LOCUS 8 in the model plant *Arabidopsis thaliana* opens up new avenues for light-controllable methodologies. In this review, we discuss current developments in optogenetic control by UV-B light and its signaling components, as well as rational considerations in the design and applications of UV-B-based optogenetic tools.

## Introduction

Plants have evolved light sensing systems to adapt to the changing ambient light environment. According to the wavelengths absorbed, three categories of photoreceptors have been characterized in plants to date. In the model dicot *Arabidopsis thaliana*, phytochromes (phyA-E) sense far-red and red light, and blue/ultraviolet-A (UV-A) light sensors include cryptochromes (cry1 and cry2), phototropins (phot1 and phot2), and Zeitlupe family members ZEITLUPE (ZTL), FLAVIN-BINDING, KELCH REPEAT, F-BOX 1 (FKF1), and LOV KELCH PROTEIN 2 (LKP2) (Kami et al. 2010). For the UV-B wavelengths (280–315 nm), UV RESISTANCE LOCUS 8 (UVR8) is a lately identified photoreceptor that mediates UV-B-induced developmental processes such as photomorphogenesis and UV-B acclimation, inhibition of thermomorphogenesis and shade avoidance, and circadian entrainment (Rizzini et al. 2011; Yin and Ulm 2017). Evolutionarily originated in chlorophytes (Han et al. 2019), UVR8 is a plant-specific photoreceptor without any exogenous chromophore (Christie et al. 2012; Wu et al. 2012), and its function is well conserved among diverse plant species (Tossi et al. 2019).

The identification of UVR8 as the UV-B photoreceptor has opened up new possibilities for light-controllable methodologies. In this review, we will discuss rational considerations in the design and applications of UV-B-based optogenetic tools, and summarize the recent developments of the optogenetic tools manipulated by UV-B light and its signaling components.

## UV-B photoreceptor UVR8

In the absence of UV-B light, UVR8 is at an inactive state as a homodimer. Upon UV-B light exposure, intrinsic tryptophan residues in UVR8 perceive the light signal, and the protein becomes photoactivated via a dimer-to-monomer switch within seconds (Christie et al. 2012; Rizzini et al. 2011; Wu et al. 2012). CONSTITUTIVELY PHOTOMORPHOGENIC 1 (COP1), a central light signaling regulator, interacts with the monomerized UVR8 within minutes, thereby initiating UV-B light signal transduction in plant cells. The physical interaction between UVR8 and COP1 relies primarily on the C-terminal VP motif of UVR8 and the C-terminal WD40 domain of COP1 (Favory et al. 2009; Lau et al. 2019; Rizzini et al. 2011). Within the UV-B signaling network, UVR8 also interacts with multiple transcription factors to mediate the development of cotyledons, hypocotyls and roots under UV-B light (Yang et al. 2018, 2020; Liang et al. 2019; Qian et al. 2020).

## Rational design of UV-B optogenetic tools

Several features of UVR8, including its inherent photoreceptor activity and its UV-B-dependent formation of complexes, can be taken advantage of in the design and application of UV-B-based optogenetic tools. (1) Unlike the other photoreceptors, UVR8 protein requires no exogenous chromophore for its light sensing activity (Christie et al. 2012; Wu et al. 2012). Thus, UVR8-based optogenetic tools can be applied even in non-plant systems without requiring synthetic chromophores. (2) The specific wavelengths of UVR8 absorption enable multicolor imaging of the fluorescent marker proteins already in common use without disturbing UVR8 photoactivation. (3) UVR8 is exceptionally sensitive to the environmental UV-B light, allowing optogenetic stimulation at light levels well below the safety threshold of cell proliferation of plant, yeast and mammalian cells (Rizzini et al. 2011). In consideration of UV-B toxicity, damage-responsive gene expression or nuclear foci formed by DNA-binding proteins can be examined as indicators of cell damage (Crefcoeur et al. 2013). (4) UV-B-induced monomerization is an intrinsic feature of UVR8, and is hardly affected by light-regulated organization of other protein complexes, as experimentally and further mathematically substantiated previously (Rizzini et al. 2011; Ouyang et al. 2014). These characteristics allow UVR8 to be a fast and robust on-switch for UV-B optogenetic regulation even under multi-chromatic light context. (5) Upon the removal of UV-B light, monomeric UVR8 reverses to the homodimeric state in vivo (Heijde and Ulm 2013; Heilmann and Jenkins 2013), enabling light switchable control. This process takes place spontaneously but slowly (over 35 h) in vitro (Wu et al. 2012), allowing tools to harness sustainable optogenetic control by a short light pulse in a system free from the factors promoting UVR8 redimerization. (6) In response to UV-B light, COP1 promotes the nuclear accumulation of UVR8 (Qian et al. 2016; Yin et al. 2016). This behavior provides the potential for strategies involving the control of protein nuclear localization by UV-B light. (7) Finally, based on UVR8 signaling pathway, tools can be designed to control successive events step by step if UVR8 monomerization, the UVR8–COP1 interaction, UVR8 nuclear accumulation and UVR8 redimerization are engineered in tandem within a system.

Current UV-B optogenetic applications mainly rely on the UV-B-induced UVR8–COP1 interaction. Actually besides COP1, a series of UVR8 interacting proteins have been identified in recent studies, including UVR8 itself, REPRESSOR OF UV-B PHOTOMORPHOGENESIS 1 (RUP1), RUP2, BRI1-EMS-SUPPRESSOR 1 (BES1), BES1-INTERACTING MYC-LIKE 1 (BIM1), WRKY DNA-BINDING PROTEIN 36 (WRKY36), MYB73, MYB77, and MYB13 (Favory et al. 2009; Gruber et al. 2010; Liang et al. 2018; Yang et al. 2018, 2020; Qian et al. 2020). Among these proteins, COP1 and MYB13/73/77 physically interact with UVR8 in a UV-B-dependent manner, enabling light-inducible application. Rather than that of the UVR8–MYB13/73/77 association, the kinetics of the UVR8–COP1 association has been extensively examined. Upon UV-B exposure, UVR8 associates with COP1 in minutes in vivo, and their tight association has been experimentally and mathematically supported (Rizzini et al. 2011; Heijde and Ulm 2013; Ouyang et al. 2014). Once induced by UV-B light pulses, their association can last for several hours (Heijde and Ulm 2013), preventing damage-related responses provoked by prolonged UV-B irradiation. Therefore, the rapid, tight and sustainable association between UVR8 and COP1 is ideal to be utilized in an optogenetic system.

Meanwhile, several crucial facts about UVR8-based optogenetic tools should be paid attention to. (1) UV-B light cannot penetrate deep enough in the tissues, limiting its applications in vivo. (2) The UVR8–COP1 association is stable but to some extent irreversible, resulting in difficulty in tool inactivation. In this case, the integration of effective switch-off modules, such as RUP1 and RUP2 which facilitate UVR8 redimerization (Heijde and Ulm 2013), may be considered to develop a switchable tool. (3) COP1 is a highly conserved E3 ubiquitin ligase in living organisms (Han et al. 2019). Though the UVR8–COP1 module is well accepted in UV-B optogenetic applications, the expression of full-length COP1 might increase the risk of unexpected substrate degradation in host cells. Therefore, a truncated form of COP1, the C-terminal 340 amino acids of COP1 (COP1^C340^) with eliminated E3 ligase activity whereas high affinity with UVR8 (Rizzini et al. 2011), is preferred in UV-B optogenetic systems. (4) Detailed determination of the association and dissociation characteristics of alternative UVR8 complexes will support more options in engineering UV-B light-controllable tools.

## Applications of UV-B optogenetic tools

The yeast two-hybrid experiments that verified the UVR8–COP1 interaction is regarded as the first example of UV-B-induced optogenetic system (Rizzini et al. 2011). Subsequently, UVR8-based optogenetic tools have been successfully applied in mammalian and yeast cells to regulate gene expression, protein localization and secretion, and chromosomal looping. Below we describe some of the systems developed to date.

### Control of gene expression

**Fig. 1:**
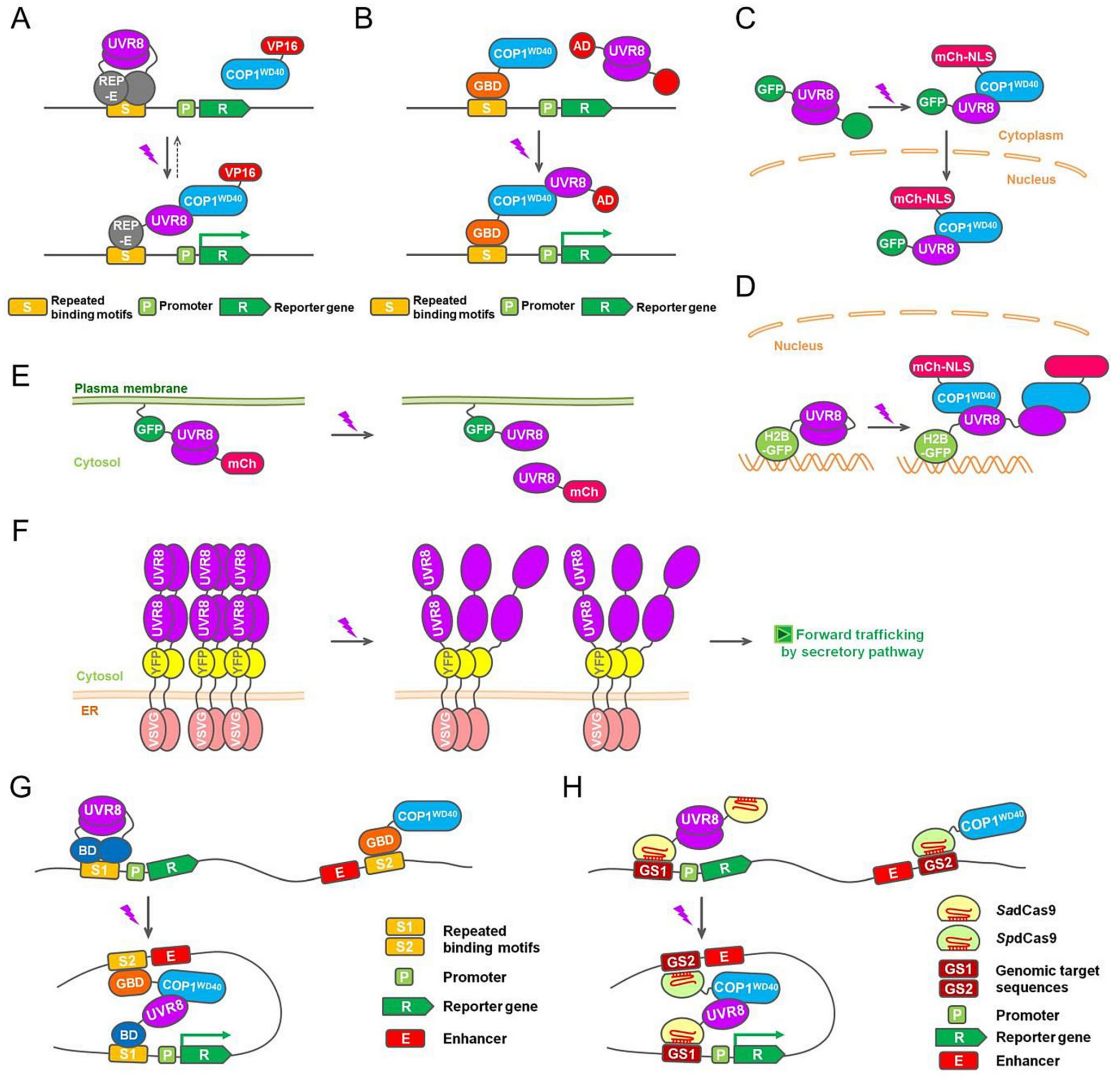
Design and applications of UV-B optogenetic tools. **A, B** UV-B light induces the activation of gene expression. **C** UV-B light induces protein translocation from cytoplasm to nucleus. **D** UV-B light induces protein association with chromatin. **E** UV-B light induces protein translocation from plasma membrane to cytosol. **F** UV-B light induces protein secretion from endoplasmic reticulum. **G, H** UV-B light induces chromosomal looping and gene expression

UV-B-inducible expression systems have been developed based on a chimeric transcription factor whose functional domains were split and, respectively, fused with UVR8 and COP1. For instance, the macrolide repressor E (REP-E) was fused with the core domain of UVR8, and the transactivation domain VP16 was fused to the WD40 domain of COP1. Treatment with UV-B light resulted in the recruitment of COP1^WD40^–VP16 to the REP-E occupied promoter, and thus the activation of the reporter gene, while darkness shut off the gene expression (Fig. 1A). The expression level of the reporter gene was subjected to quantitative analysis to build a mathematical model of kinetic expression (Muller et al. 2013). Notably, this system was not only feasible in human, monkey, hamster and mouse cells, but also coupled with blue and red light-inducible modules to differentially manipulate up to three genes in a single-cell culture, so as to achieve multi-chromatic control of an angiogenic signaling pathway (Muller et al. 2013, 2014).

In another system for gene expression activation, UVR8 and COP1^WD40^ were, respectively, fused to the transcription activation domain (AD) of NF-κB and the GAL4 DNA-binding domain (GBD). UV-B light exposure resulted in the proximity of NF-κB AD and GBD, and subsequent activation of the reporter gene (Fig. 1B) (Crefcoeur et al. 2013). This system can be performed in a visible light context. Subsecond pulses of UV-B light is efficient enough to induce gene expression which lasts for hours without the requirement of continuous UV-B exposure. Inducible expression of the proteins capable to disturb the UVR8–COP1 interaction will allow a reversible control of this system.

### Control of protein localization and secretion

To control protein localization using UV-B light, UVR8 was fused to green fluorescent protein (GFP), and COP1^WD40^ was fused to a monomeric Cherry (mCh) fluorescent protein with nuclear localization signal (NLS) in a mammalian cell system. Triggered by UV-B irradiation, cytoplasm-localized UVR8 gradually concentrated in the nucleus, based on its interaction with nucleus-localized COP1 (Fig. 1C). Moreover, UVR8 was fused to histone H2B or H3 to recruit COP1 to the chromatin (Fig. 1D). Based on this system, a UV-B LED was incorporated on a microscope, not only to set up real-time observation of protein interaction profiles within a single cell, but also to alleviate the potential for UV-B-induced damage produced by UV-B fluorescent lamps (Crefcoeur et al. 2013).

UVR8 dimerization and its excitation properties were also evaluated in a plasma membrane recruitment assay. One UVR8 was fused to GFP containing a C-terminal CaaX prenylation motif and the other to mCh. In the absence of UV-B light, UVR8 dimer was restrained on the plasma membrane due to GFP prenylation. Then, UV-B illumination immediately mediated UVR8 monomerization, and released UVR8-mCh from the plasma membrane to the cytosol (Fig. 1E) (Chen et al. 2013). This system can be also engineered for UV-B-induced protein secretion. The C-terminal intracellular domain of vesicular stomatitis virus glycoprotein (VSVG) was fused with tandem copies of UVR8. These fusion proteins formed oligomers that were sequestered in the endoplasmic reticulum (ER). A brief exposure to UV-B light triggered the dissociation of these oligomers, and rapidly led to their synchronous forward trafficking through the secretory pathway to the plasma membrane (Fig. 1F). This system was successfully applied in neuron cells to control local trafficking of secretory cargo near dendritic branch points, compatible with multicolor imaging. It will not only serve the investigation of the secretory system in morphologically complex cells such as neurons, but also be used as a screening system for effective proteins or drugs regulating secretory trafficking (Chen et al. 2013).

### Control of chromosomal looping

Three-dimensional genome organization is a recently acknowledged mechanism for accurate gene expression programming. To control gene expression mediated by complex chromosomal structure, Chromosomal Looping-based Expression Activation System in Yeast (CLEASY) has been developed based on UV-B-inducible long-range DNA interaction. This simplified eukaryotic model consisted of conditionally interacting proteins, distal transcriptional regulatory elements, and a reporter gene. Briefly, UVR8 was fused to LexA DNA-binding domain (BD), and COP1^WD40^ was fused to GAL4 DNA-binding domain (GBD). Each recombinant protein bound to one of two distal transcriptional regulatory elements (S1 and S2) integrated on a yeast chromosome. The UV-B-dependent interaction of UVR8 and COP1^WD40^ brought together the reporter promoter and its enhancer element. As a consequence, a chromosomal loop formed and the reporter gene was activated (Fig. 1G) (Qiu et al. 2021). This system was also suitable for phenotypic analyses such as cell viability assays, when related reporter genes were introduced. The design that S1 and S2 were, respectively, recognized by BD and GBD fusion proteins ensured target specificity, however, might limit target variability.

In addition to the above artificial target sequences, genomic sequences of the host cell can also be subjected to looping manipulation using this system. Combined with the clustered regularly interspaced short palindromic repeats (CRISPR)/CRISPR-associated protein 9 (Cas9) strategy, CLEASY gained both target specificity and variability. In this case, two orthogonal cleavage-defective Cas9 proteins were fused with UVR8 and COP1^WD40^ respectively. These fusion proteins formed a UV-B-induced bivalent dCas9 complex that was capable of simultaneously binding to two distal DNA segments (GS1 and GS2) via target-specific single guide RNAs, thereby mediating DNA looping and gene activation (Fig. 1H) (Qiu et al. 2021). Similarly, repression or silencing of gene expression can be programmed via long-range DNA looping in this system, determined by the function of transcriptional regulatory elements such as insulators. In short, this unicellular system can be employed as a simplified biological platform, not only for the investigation of DNA looping mechanism at a single-cell level, but also for large-scale screening of effective biomedical molecules acting on chromosome conformation organization.

## Conclusions and future perspectives

Based on plant photosensory systems, optogenetic methodologies have achieved the control of intracellular signaling pathways, and have shed light on antitumor immunomodulation (Tan et al. 2017; Zhang and Cu 2015). Despite the general success of optogenetic approaches utilizing far-red, red and blue light receptors, UV-B- or UVR8-based tools are less developed. According to the advantages and disadvantages of these tools, case-specific evaluation is a basic requirement for design and implementation of UV-B optogenetic tools. The biological context, tissue or cell type, and light dosage should be taken into account. In particular, due to weak penetrating capacity, UV-B optogenetic tools have limitations in sample targeting depth, whereas they are potentially advantageous for single-layer cells or even single-cell manipulation with target specificity and spatiotemporal precision. To overcome low penetration depth, the idea of wireless UV-B optogenetics has been proposed, with the purpose of effective light delivery and minimized invasiveness (Tan et al. 2017). A recent study in *C. elegans* has uncovered that LITE-1, a taste receptor homolog, is a bona fide photoreceptor that absorbs both UV-A and UV-B light to mediate avoidance behavior (Gong et al. 2017). The identification of additional UV-B sensory proteins will likely attract interest in their potential for optogenetic application, and increase the diversity and specificity of optogenetic tools.
